# A new classification of lymph node metastases according to the lymph node stations for predicting prognosis in surgical patients with esophageal squamous cell carcinoma

**DOI:** 10.18632/oncotarget.12842

**Published:** 2016-10-24

**Authors:** Zheng Lin, Weilin Chen, Yuanmei Chen, Xiane Peng, Kunshou Zhu, Yimin Lin, Qiaokuang Lin, Zhijian Hu

**Affiliations:** ^1^ Department of Epidemiology and Health Statistics, Fujian Provincial Key Laboratory of Environment Factors and Cancer, School of Public Health, Fujian Medical University, Fuzhou 350108, China; ^2^ Department of Radiation Oncology, Zhangzhou Affiliated Hospital of Fujian Medical University, Zhangzhou 363000, China; ^3^ Department of Thoracic Surgery, Fujian Provincial Cancer Hospital Affiliated to Fujian Medical University, Fuzhou 350014, China; ^4^ Key Laboratory of Ministry of Education for Gastrointestinal Cancer, Fujian Medical University, Minhou, Fuzhou 350108, China

**Keywords:** LNM, prognosis, ESCC, RSF

## Abstract

Lymph node metastasis (LNM) is one of the major prognostic factors for esophageal squamous cell carcinoma (ESCC). However there is no consensus regarding the prognostic significance of the location of LNM. Therefore, a novel classification was proposed to identify the lymph node (LN) stations which may be useful in predicting prognosis. A total of 260 ESCC patients were enrolled in this prospective study. The prognostic values of LNM in different lymph node (LN) stations were evaluated by random survival forests (RSF). Their prognostic significance was examined by Cox regression and receiver operating characteristic curve (ROC). The three most frequently involved LN stations were station 16 (24.49%), station 1 (22.22%) and station 2 (21.05%). Stations 1, 2, 8M, 8L and 16 were grouped as dominant LN stations (DLNS) which showed higher values in predicting overall survival (OS) and disease-free survival (DFS) than the remaining LN stations, which we define as non-dominant LN stations (N-DLNS). LNM features of DLNS (number of positive LN stations, number of positive LNs and LN ratio), but not those from N-DLNS, served as independent prognostic factors (P<0.05) whenever used alone or when combined with factors from N-DLNS. Furthermore, the area under ROC indicated that DLNS is a more accurate prediction than N-DLNS (P<0.05). This study demonstrated the value of LNM in DLNS in predicting prognosis in surgical ESCC patients, which outperformed those from N-DLNS. Therefore, the method of dominant and non-dominant classification may serve as an additional parameter to improve individualized therapeutic strategies.

## INTRODUCTION

Lymph node metastasis (LNM) is one of the most important prognostic factors for esophageal squamous cell carcinoma (ESCC). The 7^th^ edition of the TNM staging system, published by the American Joint Committee on Cancer (AJCC) [1] and the Union for International Cancer Control (UICC), [2] uses the number of metastatic regional lymph nodes (LN) as one criterion for N staging. The supporting evidence cited for the adoption of this un-anatomic N classification is the lack of a difference in survival rate with respect to the various involved nodal groups, suggesting that these lymph nodes stations should be staged equivalently [3, 4].

Some researchers aiming to optimize the lymphadenectomy during esophagectomy for better survival, hypothesized that the metastatic regional nodal groups, which are located in different anatomical zones, may not share equal prognostic significance [5–16]. Advocates of this theory argue that it is necessary to consider the location of a lesion, as found in the Japanese Society for Esophageal Disease (JSED) N staging system [17]. Since LNM patterns shift according to tumor location [18–20], and the extent of LNM is closely associated with the depth of cancerous invasion and tumor location [17, 21], it is reasonable to suggest that the prognostic significance of LNM occurring in specific LN stations would vary by tumor location. However, studies evaluating the prognostic values of specific metastatic nodal regions, such as cervical [8–10, 14], recurrent laryngeal [11–14], subcarinal [15, 16], and pre-tracheal [15, 16], yielded inconsistent results. This discordance existed even in populations with similar depth of lesion invasion [22, 23] or distribution of tumor location [12, 14].

The lack of consensus regarding the prognostic significance of the location of LNM, casts doubt on the utility of N stage in improving individualized therapeutic strategies [24]. Due to the fact that multi-station LN involvement [25] and skipped metastases [26] were frequently observed in ESCC patients, it is difficult to evaluate the prognostic value of LNM separately based on anatomical zone. The purpose of this study was to categorize the metastatic LN stations as dominant and non-dominant groups according to their relative prognostic importance, and to examine the feasibility and utility of this classification method in predicting the prognosis of ESCC patients.

## RESULTS

### Patient characteristics and the results of follow-up

Demographic, clinical and pathological characteristics of the 260 ESCC patients enrolled during the study period are listed in Table 1. The median age of patients was 61 years. The majority of patients were male (n=201, 77.3%) and the middle thoracic esophagus (MTE) was most often involved (n=173, 66.5%). Nodal metastasis was detected in 141 patients (54.2%). Tumor stage classification (TNM) showed 51.5% (n=133) of them were stage III cases, but none with distant metastasis (all patients were M0). The median value of harvested lymph node (HLN) was 35, with lower and upper quartile 25 and 46, and the median number of positive lymph node (PLN) was 1 with interquartile range 3.

**Table 1:** Descriptions of demographic, clinical and pathological characteristics for the 260 patients included in this study

Characteristic	Total (n=260)	
	n	%
Age (year)		
Median (P_25_, P_75_)	61 (52, 67)	
Sex		
Male	201	77.3
Female	59	22.7
Tumor location		
CE/UTE	28	10.8
MTE	173	66.5
LTE	59	22.7
Tumor length (cm)		
Median (P_25_, P_75_)	4.0 (3.0, 4.5)	
Primary tumor (pT)		
pT1	30	11.5
pT2	44	16.9
pT3	164	63.1
pT4	22	8.5
Regional lymph nodes (pN)		
pN0	119	45.8
pN1	67	25.1
pN2	54	20.8
pN3	20	7.7
Distant metastasis (M)		
M0	260	100
M1	0	0
Histologicgrade (pG)		
pG1	79	30.4
pG2	148	56.9
pG3	18	6.9
Tumor stage (pTNM)		
0	3	1.2
IA	7	2.7
IB	28	10.8
IIA	37	14.2
IIB	52	20.0
IIIA	61	23.5
IIIB	37	14.2
IIIC	35	13.5
LVI		
Yes	71	27.3
No	189	72.7
PNI		
Yes	62	23.8
No	198	76.2
Type of lymphadenectomy		
3-FLND	153	58.8
2-FLND	107	41.2
Residual tumor (R)		
Rx	5	1.9
R0	251	96.5
R1	4	1.5
Anastomotic leak		
Yes	52	20.0
No	191	73.5
Postoperative infection*		
Yes	124	47.7
No	119	45.8
Chemotherapy		
Yes	74	28.5
No	186	71.5
Radiotherapy		
Yes	59	22.7
No	201	77.3
Harvested lymph nodes (HLNs)		
Median (P_25_, P_75_)	35 (25, 46)	
Positive lymph nodes (PLNs)		
Median (P_25_, P_75_)	1 (0, 3)	

CE, cervical esophagus; UTE, upper thoracic esophagus; MTE, middle thoracic esophagus; LTE, lower thoracic esophagus; LVI, lymphovascular invasion; PNI, perineural invasion; 3-FLND, three-field lymph node dissection; 2-FLND, two-field lymph node dissection.

The median follow-up duration (MFD) for overall survival (OS) and disease-free survival (DFS) were 1040 days and 963 days, respectively. In the 3-year period, the cumulative overall and disease-free survival rates were 53% and 45%, respectively.

### LNM patterns in ESCC patients

The three most frequently involved LN stations were paracardial nodes (station 16, 24.49%), supraclavicular nodes (station 1, 22.22%), and upper paratracheal nodes (station 2, 21.05%) (Table 2). LNM rates were evaluated by tumor location. The three most frequently involved LN stations for cervical esophagus/upper thoracic esophagus (CE/UTE) cases were station 1 (38.89%), station 2 (29.27%), and station 9 (25%). Station 8M (23.36%), station 1 (22.86%), and station 16 (22.84%) were the three most frequently involved for middle thoracic esophagus (MTE) cases. Station 16 (33.9%), station 8L (26.67%), and station 8M (17.5%) were the most frequently involved for lower thoracic esophagus (LTE) cases. No significant difference of LNM incidence was detected between the three tumor locations; however, the metastatic rate in supraclavicular nodes tended to decrease if the tumor location changed from the lower to the upper part of the esophagus (*P*_trend_=0.023, LN Station 1 Table 2). The reverse trend was observed in paracardial nodes (*P*_trend_ =0.035, LN Station 16 Table 2).

**Table 2:** Comparisonsof LNM incidence in specific regional lymph node stations across CE/UTE, MTE and LTE

LN station	CE/UTE (n=28)			MTE (n=173)			LTE (n=59)			Total (n=260)			Exact P Value ^a^	Trend P Value^b^
	Number of patients with PLN	Number of patients with HLN	Prevalence of LNM (%)	Number of patients with PLN	Number of patients with HLN	Prevalence of LNM (%)	Number of patients with PLN	Number of patients with HLN	Prevalence of LNM (%)	Number of patients with PLN	Number of patients with HLN	Prevalence of LNM (%)		
1	7	18	38.89	24	105	22.86	3	30	10.00	34	153	22.22	0.066	0.023*
2	7	24	29.17	32	143	22.38	5	42	11.90	44	209	21.05	0.174	0.093
3p	2	18	11.11	12	105	11.43	0	24	0.00	14	147	9.52	0.204	0.196
4	2	11	18.18	8	88	9.09	1	22	4.55	11	121	9.09	0.358	0.351
5	0	1	0.00	2	21	9.52	0	6	0.00	2	28	7.14	1.000	1.000
6	0	1	0.00	1	10	10.00	0	3	0.00	1	14	7.14	1.000	1.000
7	1	23	4.35	17	141	12.06	5	49	10.20	23	213	10.80	0.662	0.700
10	0	19	0.00	17	145	11.72	3	49	6.12	20	213	9.39	0.223	1.000
8M	2	17	11.76	25	107	23.36	7	40	17.50	34	164	20.73	0.509	1.000
8L	2	14	14.29	21	116	18.10	12	45	26.67	35	175	20.00	0.444	0.233
9	1	4	25.00	6	39	15.38	2	17	11.76	9	60	15.00	0.708	0.744
15	0	4	0.00	1	35	2.86	1	14	7.14	2	53	3.77	0.568	0.528
16	3	24	12.50	37	162	22.84	20	59	33.90	60	245	24.49	0.088	0.035*
17	2	17	11.76	12	81	14.81	7	37	18.92	21	135	15.56	0.783	0.564
18	0	9	0.00	4	65	6.15	0	26	0.00	4	100	4.00	0.710	0.662
19	0	4	0.00	1	22	4.55	0	8	0.00	1	34	2.94	1.000	1.000
20	0	4	0.00	1	19	5.26	0	10	0.00	1	33	3.03	1.000	1.000

CE, cervical esophagus; UTE, upper thoracic esophagus; MTE, middle thoracic esophagus; LTE, lower thoracic esophagus; PLN, positive lymph node; HLN, harvested lymph node; LNM, lymph node metastasis;

a 2-sided Fisher's exact test for the incidence of PLN between three different locations

b 2-sided test of linear by linear association between the location (as ordinal variable) and incidence of LNM

* P<0.05.

**Figure 1:**
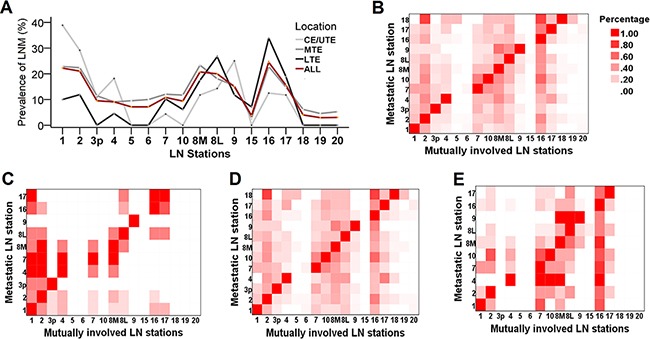
The pattern of lymph node metastasis (LNM) in regional nodal stations **A.** Multiple line chart of incidence of LNM in varying nodal stations separated by tumor location. The light gray, dark gray, black and dark red lines indicate tumor was located at CE/UTE, MTE, LTE and all locations, respectively. No significant difference of incidence of LNM was observed at any LN stations for the three locations. **B.** The pattern of multiple lymph node metastatic involvement in all patients, and separated by tumor locations **C.** CE/UTE, **D.** MTE and **E.** LTE. Darker color indicates the prevalence in the specific LN station is high, while the lighter color shows low prevalence.

Similar LNM patterns were detected in CE/UTE, MTE and LTE ESCC patients (Figure 1A), and multi-station metastases were found in 82 patients (58.2% of all LNM cases). Heat mapping was applied to demonstrate the multi-LNM distribution pattern (Figure 1B-1E). For example, when LNM occurred at station 1, high chances of mutual involvement were also observed at stations 2, 7 and 16 (Figure 1B). Multi-station metastases appeared to vary according to tumor locations. LN stations concurrently involved in CE/UTE cases include cervical (station 1) and upper paratracheal nodes (station 2) (Figure 1C). While for tumors located at LTE, the hot area was the mid/lower mediastinum (stations 7, 10, 8M, 8L and 9) or upper abdomen nodes (station 16 and 17) (Figure 1E). However, for MTE cases, obvious tendencies to develop bidirectional multi-station metastases, cervical (station 1 and 2) and abdominal nodal zones (station 16 and 17) were equally frequently involved (Figure 1D).

### Impacts of nodal metastasis on prognosis

**Figure 2:**
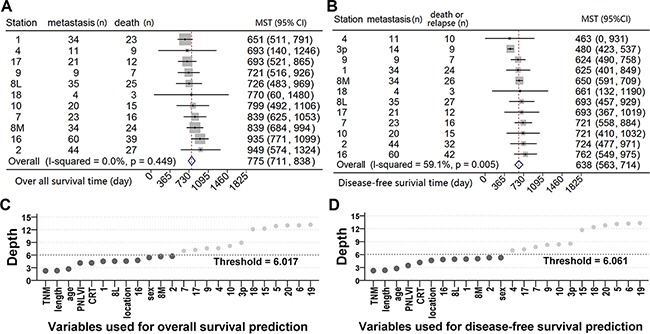
The impact of nodal metastasis on survival Forests plot of 95% confidence intervals of median survival time (MST) of **A.** overall and **B.** disease-free survival. The points inside the gray boxes represent the point estimates of the MST, the size of each box is proportionate to the weight of each nodal station, and the horizontal bars denote the 95% CI of MST. Two random survival forest (RSF) models were used for hunting for the important variables associated with **C.** poor overall and **D.** disease-free survival. The variables with depth below the thresholds will be selected as the important variables (dark gray dots).

Data from metastatic ESCC patients were grouped according to the involved LN stations, and the median survival time (MST) and 95% confidence intervals (CI) were calculated and plotted in Figure 2A and Figure 2B. The forest plot of DFS showed moderate heterogeneity (I^2^=59.1%, *P*=0.005, Figure 2B). Therefore, we concluded that metastasis in different LN stations may affect the prognosis in a heterogeneous manner.

Thus, two separate random survival forests (RSF) models were constructed with the same sets of candidate variables, and used to identify important factors associated with overall and disease-free survival (Supplementary Table S2). The scatter plot shows that TNM stage, tumor length, age, perineural/lymphatic/vascular invasion (PNLVI), chemoradiotherapy (CRT), tumor location, and sex, as well as the metastatic status of several LN stations were factors for predicting overall (Figure 2C) and disease-free survival (Figure 2D). Since stations 1, 2, 8M, 8L and 16 were qualified in both models, they will be grouped as dominant lymph node stations (DLNS), while the other 12 regional LN stations will be called non-dominant lymph node stations (N-DLNS).

### Metastases in DLNS serve as a strong prognostic factor and are associated with poorer survival than metastases in N-DLNS

We excluded 13 cases with HLN<16 in the Kaplan-Meier survival analysis and Cox regression to reduce potential interference from inaccurate staging in our results. The remaining cases were categorized into the following four groups according to the LNM status: nodes negative patients (pN0, n=109), metastasis in N-DLNS only (N-DLNS+, n=15), metastasis in DLNS only (DLNS+, n=67) and both positive (both+, n=56).

**Figure 3:**
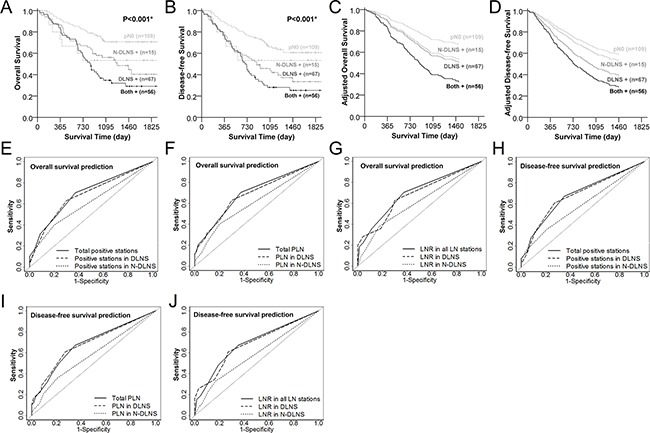
Lymph nodes metastasis in dominant lymph node stations (DLNS) serves as a stronger predictor to poorer overall and disease-free survival than non-dominant lymph node stations (N-DLNS) Overall survival **A.** and disease-free survival curves **B.** of patients with DLNS and/or N-DLNS metastasis. Light gray solid, dark gray dashed, dark gray solid and black solid lines represent the pN0, N-DLNS positive only, DLNS positive only, and both positive cases, respectively. The Cox regression adjusted survival functions plotted in **C.** (overall survival)and **D.** (disease-free survival) were adjusted for 60-year old male, tumor located at middle thoracic esophagus with length of tumor 3.8 cm, adventitia invasion (pT3), without PNLVI and chemoradiotherapy. ROC curves of variables from DLNS and N-DLNS served as predictors for 4-year overall **E, F**, and **G.** or disease-free survival **H, I** and **J.** Solid, dashed and dotted lines represent the indicators from total, DLNS, and N-DLNS, respectively.

The DLNS+ group had poorer OS (*P*=0.002) and DFS (*P*<0.001) than the pN0 group (Figure 3A and 3B). However, there was no evidence for poorer DFS (*P*=0.064) in N-DLNS+ cases compared to pN0 cases. The survival curves generated by the Cox regression modeling for the association of LNM in DLNS and N-DLNS with prognosis, Table 3, also demonstrate a poorer prognosis for DLNS+ groups than for N-DLNS+ groups (Figure 3C and 3D). Furthermore, the model illustrates that N-DLNS+ did not indicate poorer survival when compared with pN0 cases (*P*=0.348, *P*=0.714 for OS and DFS, respectively), while DLNS metastases reduced the survival rate (for OS, HR=1.720, 95% CI=1.017-2.911, *P*<0.001; for DFS, HR=1.767, 95% CI=1.099-2.840, *P*=0.001).

**Table 3:** Multivariate Cox regression results of association of lymph node metastasis (LNM) in dominant lymph node stations (DLNS) and non-dominant lymph node stations (N-DLNS) with prognosis

Model^a^	Variables	Overall Survival			Disease-free Survival		
		HR	95% CI of HR	P Value	HR	95% CI of HR	P Value
1^b^	pN0 (n=109)	—	—	—	—	—	—
	N-DLNS(+) only (n=15)	1.593	0.602-4.214	0.348	1.195	0.460-3.103	0.714
	DLNS(+) only (n=67)	1.720	1.017-2.911	0.043*	1.767	1.099-2.840	0.019*
	Both(+) (n=56)	2.852	1.635-4.973	<0.001*	2.383	1.417-4.008	0.001*
			<0.001 (trend)*			0.001 (trend)*	
2^c^	Number of (+) stations in DLNS	1.270	1.007-1.601	0.043*	1.247	1.007-1.545	0.043*
	Number of (+) stations in N-DLNS	1.220	0.950-1.566	0.119	1.118	0.877-1.426	0.369
3^c^	Number of PLNs in DLNS	1.092	1.011-1.180	0.025*	1.101	1.021-1.187	0.012*
	Number of PLNs in N-DLNS	1.009	0.871-1.169	0.903	0.964	0.832-1.117	0.625
4^c^	LNR^d^ in DLNS	1.582	1.039-2.407	0.032*	1.490	1.007-2.205	0.046*
	LNR^d^ in N-DLNS	1.250	0.889-1.738	0.185	1.068	0.778-1.467	0.682
5^c^	Number of (+) stations in DLNS	1.331	1.063-1.668	0.013*	1.277	1.037-1.573	0.021*
6^c^	Number of PLNs in DLNS	1.094	1.022-1.173	0.010*	1.092	1.021-1.169	0.011*
7^c^	LNR^d^ in DLNS	1.648	1.080-2.515	0.021*	1.497	1.010-2.218	0.044*
8^c^	Number of (+) stations in N-DLNS	1.300	1.022-1.654	0.032*	1.187	0.937-1.503	0.155
9^c^	Number of PLNs in N-DLNS	1.078	1.039-1.236	0.280	1.031	0.896-1.185	0.674
10^c^	LNR^d^ in N-DLNS	1.315	0.938-1.842	0.112	1.083	0.781-1.503	0.633

a Excludes 13 cases (5%) with HLN<16, 10 cases from pN0, 2 cases from N-DLNS(+), and 1 case from DLNS(+) group.

b Age (as continuous), sex (female, male), location of tumor (upper, middle, lower), length of tumor (as continuous), pT (I or II, III or IV), PNLVI (no, yes) and chemoradiotherapy (no, yes) were included as covariates in Model 1.

c Models 2 to 10 shared the same covariates (age (as continuous), sex (female, male), location of tumor (upper, middle, lower), length of tumor (as continuous), PNLVI (no, yes), TNM (stage I or II, stage III) and chemoradiotherapy (no, yes)).

d LNR was categorized into 3 levels (LNR=0, 0<LNR<20% and LNR≥20%).

* P<0.05.

To further depict the impact of the DLNS and N-DLNS metastasis on prognosis, the number of positive stations, number of positive lymph nodes (PLNs) and lymph node ratios (LNR) from DLNS and N-DLNS were evaluated by Cox regression (Table 3). The indicators of DLNS showed significant in all models for predicting survival (Models 2 to 7, Table 3). However, the indicators of LNM in N-DLNS failed to predict survival (Models 2, 3, 4, 9 and 10, Table 3).

Finally, receiver operating characteristic (ROC) curves were used to evaluate the potential prediction effectiveness in overall and disease-free survival by metastatic indicators of DLNS and N-DLNS. All of the indicators of DLNS (number of positive LN stations, PLN and LNR) offered greater effectiveness for predicting the 4-year DFS than the indicators of N-DLNS (*P=*0.012 for number of positive stations, *P*=0.004 for number of PLN and *P*=0.005 for LNR, Table 4). So did the number of PLN and LNR from DLNS for predicting the 4-year OS (*P*=0.043 for PLN and *P*=0.047 for LNR, Table 4). The ROC curves of DLNS overlapped those derived from whole LN stations, and the DLNS curves embraced the N-DLNS curves in all six plots (Figure 3 panels E, F, and G show 4 year overall survival. Panels H, I, and J show4 year disease-free survival.).

**Table 4:** Comparisons of ROC curves between DLNS and N-DLNS metastasis for 4-year survival

Survival	Predictor	Number of positive stations			Number of PLN			LNR^a^		
		AUC (%)	SE (%)	P	AUC (%)	SE (%)	P	AUC (%)	SE (%)	P
OS	DLNS	68.24	3.59	0.062	68.27	3.57	0.043*	68.14	3.56	0.047*
	N-DLNS	60.96	3.27		60.43	3.34		60.58	3.34	
DFS	DLNS	68.01	3.56	0.012*	68.64	3.29	0.004*	68.32	3.47	0.005*
	N-DLNS	58.63	3.19		58.03	3.25		58.11	3.29	

OS, overall survival, DFS, disease-free survival; DLNS, dominant lymph node stations; N-DLNS, non-dominant lymph node stations; PLN, positive lymph node.

a LNR were categorized into 6 levels (LNR=0, 0<LNR<5%, 5%≤LNR<10%, 10%≤LNR<15%, 15%≤LNR <20% and LNR≥20%).

* P<0.05.

### The dominant prognostic value of DLNS may be due to higher LNR compared to N-DLNS

To explore possible reasons underlying the greater prognostic values of DLNS over N-DLNS, we compared the demographic, clinical, and pathological features between cases with metastasis exclusively located at DLNS and those exclusively at N-DLNS (Supplementary Table S3). However, no significant differences were detected in these traits between these two groups (all *P*>0.05, Supplementary Table S3).

In this study, the surgeons tended to resect more LNs in DLNS than N-DLNS when LNM was confirmed (*P*<0.001, Table 5), which may contribute to the detection of more positive LN stations (*P*<0.001, Table 5) and PLNs (*P*<0.001, Table 5) in DLNS than in N-DLNS. However, significantly higher LNR were also observed in DLNS than in N-DLNS (*P*<0.001, Table 5). Since the LNR is the ratio of PLNs to HLNs, the greater prognostic value of DLNS may partly attribute to higher chances of LNM.

**Table 5:** Comparisons of the features of lymphadenectomy and lymph node metastasis between DLNS and N-DLNS in 138 N+ ESCC patients^a^

Features^b^	DLNS	N-DLNS	P^c^
Number of HLN	20 (15, 27)	16 (10, 22)	<0.001*
Number of positive stations	1 (1, 2)	1 (0, 1)	<0.001*
Number of PLN	2 (1, 4)	1 (0, 2)	<0.001*
LNR (%)	9.3 (4.8, 17.6)	2.8 (0.0, 12.5)	<0.001*

HLN, harvested lymph nodes; PLN, positive lymph nodes; LNR, lymph node ratio

a Exclude the case with HLN<16.

b The statistical description of these features were expressed as median (lower quartile, upper quartile).

c Paired-samples Wilcoxon sign rank test P value.

* P<0.05.

## DISCUSSION

In this prospective study, we proposed a novel approach to categorize regional LN stations of ESCC into DLNS and N-DLNS. Supraclavicular (stations 1), paratracheal (station 2), middle and lower paraesophageal (station 8M and 8L), as well as paracardial (station 16) nodes were grouped as DLNS according to their higher prognostic importance, while the other LN stations served as N-DLNS. Metastases involved in DLNS were better at predicting survival, therefore the metastasis features of DLNS may serve as an intuitive and simple index to evaluate prognosis in surgical ESCC patients.

In this study, the prevalence of LNM varied depending on tumor location. According to Niwa Y, et al. [27], using similar surgical procedures, the LNM rates were close to our results when separated by tumor location. Multiple LNM were frequently observed in our study, and this phenomenon was also observed by other researchers. In the study of Hsu PK, et al. [10], the LNM at right upper mediastinum correlated with the neck, upper/middle third esophagus, and abdominal nodal groups. Tabira Y, et al. [14] also found that recurrent nerve nodal involvement was associated with cervical nodal metastasis. Moreover, the heat mapping of different tumor locations indicated that multiple LN metastases patterns may shift according to the position of the lesion. This shift was consistent with the results reported by Bin L, et al. [18]. In their research, cervical LNM was more common in patients with a tumor located in the upper part of the esophagus, and abdominal LNM was more frequent in patients with tumor situated in the lower part of the esophagus. In addition, the more obvious tendency of bidirectional metastatic patterns from MTE was also reported by Chen J, et al. [19] and Akiyama H, et al [25].

Tumor location [17, 18] and depth of lesion [21] both influence the extent of LNM, which could partly explain the inconsistency between the studies which focused on evaluating the prognostic values of nodal metastases. However, even in the case of studies which had similar proportions of depth of lesion invasion or distribution of tumor location, the results were not in agreement when assessing the prognostic significance of the cervical [22, 23] or recurrent nerve [12, 14] LN metastases. Furthermore, multi-station LNM were frequently detected in ESCC patients [25], which also hindered evaluation of the prognostic value of LNM in specific LN stations or in LN zones separately.

Therefore, we proposed that some LN stations should be merged according to their relative importance to their association with survival and considered as an entirety. Indexes which indicate LNM in DLNS, whether used alone or combined with indicators from N-DLNS, were verified for their prognostic values in predicting OS and DFS. However, those indicators from N-DLNS failed to predict prognosis. Moreover, the AUC of ROC confirmed the predominance of DLNS. Therefore, we concluded that the prognostic role of LNM is predominantly introduced by DLNS.

Although the concept of DLNS is anatomically independent, it is still plausible to explain the connections between relatively distant LN stations. The presence of abundant long longitudinal lymphatic drainage in the submucosa facilitate the spread of cancer cells to distant LNs, even bypassing the LNs located close to a primary tumor. Therefore stepwise and skipped metastasis are both common in ESCC. This intramural and longitudinal, rather than segmental, nature of the esophagus lymphatic draining network [28], allowed us to connect relatively distant LN stations and group them into DLNS.

It was reported that HLNs were positively associated with PLNs [29] or the probability of detecting PLNs [30], thus relatively lower HLN will decrease the chance of PLN detection. As shown in our results, the median of HLN in DLNS was significantly higher than N-DLNS, therefore more extensive lymphadenectomy could be partly responsible for the predominant effect of DLNS. However, the LNR in DLNS was also significantly higher than in N-DLNS. Because LNR was defined as the ratio of PLNs to HLNs [31], this index could diminish the impact of inadequate LN resection when applied to evaluation of nodal metastases' prognostic values [32]. Moreover, previous research has verified LNR as an independent prognostic factor in predicting survival for ESCC patients [32, 33]. Therefore, the predominant value of DLNS may be attributed to relatively higher LNR in these nodal groups.

There are two limitations in this study. First, this is a single institutional study and the patients were enrolled from a medium-sized hospital, which may make the results from this study not generalizable to other populations. However, our study population came from the hospital most well known in the area for treatment of ESCC, providing a large number of ESCC patients. Single-sourced inclusion will minimize the chance of surgeon's preference for lymphadenectomy.

The second limitation is that we studied relatively few upper thoracic esophagus (UTE) cases, which could affect our conclusions. In a recently published large scale study, the proportion of UTE cases was 8.8%[34] which was close to our proportion of cases (10.8%). The LNM pattern in UTE from our study was similar to a previous report [27] which reported cases collected for 10 years. Moreover, the application of the random survival forests approach could partly reduce the instability during modeling.

In summary, our study showed that the metastatic statuses of DLNS may be useful in predicting prognosis in surgical ESCC patients, potentially serving as an additional reference for a better individualized therapeutic strategy. Multi-center or large scale studies are needed to further investigate the prognostic value of DLNS and explore molecular mechanisms of lymph node metastasis.

## MATERIALS AND METHODS

### Patients

The patients enrolled in our study underwent curative esophagectomy between December 2009 and March 2013 at the department of Thoracic Surgery, Zhang Zhou Hospital, in Fujian Province, China. All patients received preoperative endoscopic esophageal ultrasound (EUS) and an esophagoscope biopsy followed by pathological diagnosis. Esophageal carcinoma cases meeting the following criteria were excluded from our study: (1)non-squamous cell carcinoma; (2) underwent pre-operative chemotherapy or radiotherapy; (3) distant metastasis; (4)number of harvested LN (HLN) less than 6; (5)non-primary esophageal carcinoma. A total of 260 consecutive ESCC patients were included in our cohort.

Baseline demographic information for ESCC patients was collected on the date of hospital admission. The clinical and pathological traits were recorded during the hospitalization, and the postoperative radiotherapy and/or chemotherapy status was also documented. Tumor location, primary tumor (T stage), regional LNs (N stage), and histological grade (G stage) were coded according to the 7th edition of AJCC cancer staging manual [1]. The details of LN stations are listed in Supplementary Table S1. Disease relapse was diagnosed by EUS and computed tomography (CT) during postoperative follow-up.

This study was approved by the Ethics Committee of Fujian Medical University.

### Follow-up

The last follow-up was conducted in July 2015. A standard strategy of follow-up was adopted. Periodical clinical examination records inspections or telephone interviews were used to trace the patients. All patients were followed every 3 months in the first 2 years of the post-operation period and every 6 months thereafter. All death information was confirmed by contacting the patient's family or retrieving the information from the local mortality registration department.

The date of death, disease relapse or the last successful contact was recorded as the date of last follow-up. Survival time in OS was defined as the interval between the date of operation and date of death. For DFS, survival time was interpreted as the interval between the date of operation and the date of either disease relapse or death whichever came first. Patients who were still alive at the end of the follow-up or with whom contact had been lost were coded as censored.

### Surgical and lymphadenectomy procedures

The radical tri-incisional (right neck, left posterolateral, and abdominal) esophagectomy (McKeown-type) was chosen as the primary surgical therapy procedure. Three-field lymph node dissection (3-FLND) was adopted for the patients who met following criteria: cervical lymphadenectasis detected by ultrasonography with a short radius >1cm or ratio of short to long radius larger than 0.8. For cases without evidence of cervical lymphadenectasis after EUS or CT examination, a thoracoabdominal two-field lymph node dissection was performed.

All harvested lymph nodes (HLN) were recorded according to the AJCC regional LN definition [1], and were submitted for metastatic LN detection. The positive LNs (PLN) were confirmed by a trained pathologist using haematoxylin eosin (HE) staining.

### Statistical analysis

Prevalence of LNM and lymph nodes ratio (LNR) were calculated according to the following formulas:

$$\text{Prevalence of LNM}=\frac{\text{Number of patients with LNM}}{\text{Number of patients receiving lymphadenetomy}}$$

and

$$\text{LNR}=\frac{\text{Number of PLN}}{\text{Number of HLN}}$$

Fisher's exact tests were performed to evaluate the differences of prevalence of LNM between tumor locations. Survival functions were estimated by the Kaplan-Meier method. Log-rank tests were performed to compare the differences of survival rates. The extent of heterogeneity of median survival time (MST) of specific nodal metastasis was evaluated with Q statistic and I^2^ [35]. A minimal depth algorithm [36] was used to search for the important variables associated with OS and DFS using random survival forests (RSF) [37] modular in R (random Forest SRC modular). Age, sex, location of tumor, length of tumor, pathological TNM classifications, perineural/lymphatic/vascular invasion (PNLVI), postsurgical chemoradiotherapy (CRT), and the metastasis status of 17 LN stations were included in the RSF. This model was composed of 8000 trees with additional arguments using their default criteria.

The survival rates in DLNS and N-DLNS groups were compared by Log-rank test. Before performing the multivariate Cox regression test, the variables identified by the RSF model were investigated for collinearity, and the variance inflation factor (VIF) threshold was set to 3. The proportional hazards assumption was assessed by statistical test using the Schoenfeld residuals [38].

The predictive values of metastasis in DLNS and N-DLNS associated with prognosis were expressed as the area under curve (AUC), which was calculated by the time-dependent receiver operating characteristic curve (ROC) in R (time ROC modular) [39]. The paired-samples Wilcoxon sign rank test was used to compare the features of lymphadenectomy and lymph node metastasis between DLNS and N-DLNS.

The majority of statistical analyses were conducted with SAS v9.2 (Stata Corp LP, College Station, TX). All statistical tests were 2-tailed, and P≤0.05 values were interpreted as statistically significant.

## SUPPLEMENTARY TABLES




