# Supplementary material for: A new classification of lymph node metastases according to the lymph node stations for predicting prognosis in surgical patients with esophageal squamous cell carcinoma
Source: Oncotarget. 2016 Oct 24;7(46):76261–73. doi: 10.18632/oncotarget.12842 (PMC5342812; doi:10.18632/oncotarget.12842)
Supplement: Supplementary file 1 [file oncotarget-07-76261-s001.pdf]

A new classification of lymph node metastases according to the lymph node stations for predicting prognosis in surgical patients with esophageal squamous cell carcinoma

SUPPLEMENTARY TABLES

Supplementary Table S1: LN stations codes and the corresponding AJCC regional nodal stations

Stations codes	AJCC regional LN stations	Stations codes	AJCC regional LN stations
1	Supraclavicular	9	Pulmonary ligament
2	Left or right upper paratracheal	10	Left or right tracheobronchial
3P	Posterior mediastinal	15	Diaphragmatic
4	Left or right lower paratracheal	16	Paracardial
5	Aortopulmonary	17	Left gastric
6	Anterior mediastinal	18	Common hepatic
7	Subcarinal	19	Splenic
8M	Middle paraesophageal	20	Celiac
8L	Lower paraesophageal		

Supplementary Table S2: Variables selected for predicting prognosis in CE/UTE, MTE and LTE patients.

See Supplementary File 1

Supplementary Table S3: Comparison of demographic, clinical and pathological features between cases with LNM in DLNS only and those with N-DLNS only

Characteristic	N-DLNS + (n=17)		DLNS +(n=68)		P
	n	(%)	n	(%)	
Age (years)					
Median (P_{25}, P_{75})	59	(56, 65)	61	(54,68)	0.617[a]
Tumor length (cm)					
Median (P_{25}, P_{75})	3	(3, 5)	4	(3, 5)	0.193[a]
sex					
male	11	64.7	53	77.9	0.345[b]
female	6	35.3	15	22.1	
Tumor location					
CE/UTE	2	11.8	6	8.8	0.674[b]
MTE	13	76.5	47	69.1	
LTE	2	11.8	15	22.1	
pT					
pT1	1	5.9	1	1.5	0.671[b]
pT2	3	17.6	12	17.6	
pT3	11	64.7	49	72.1	
pT4	2	11.8	6	8.8	
pN					
pN1	12	70.6	48	70.6	1.000[b]
pN2	5	29.4	18	26.5	
pN3	0	0.0	2	2.9	
pG[c]					
pG1	2	12.5	20	30.8	0.308[b]
pG2	13	81.2	40	61.5	
pG3	1	6.3	5	7.7	
pTNM					
II	3	17.6	10	14.7	0.718[b]
III	14	82.4	58	85.3	
PNLVI					
no	11	64.7	36	52.9	0.426[b]
yes	6	35.3	32	47.1	
CRT					
no	8	47.1	35	51.5	0.792[b]
yea	9	52.9	33	48.5	

[a]Two independent samples Mann-Whitney U test.
[b] Fisher's exact test.
[c]1 case with missing pG information in N-DLNS metastasis group.
