# Supplementary material for: A new classification of lymph node metastases according to the lymph node stations for predicting prognosis in surgical patients with esophageal squamous cell carcinoma
Source: Oncotarget. 2016 Oct 24;7(46):76261–73. doi: 10.18632/oncotarget.12842 (PMC5342812; doi:10.18632/oncotarget.12842)
Supplement: Supplementary file 2 [file oncotarget-07-76261-s002.docx]

**Table S2.Variables selected for predicting prognosis in CE/UTE, MTE and LTE patients.**

| Survival | CE/UTE (n=28) | |  | MTE (n=173) | |  | LTE (n=59) | |  | All locations (n=260) | |
| --- | --- | --- | --- | --- | --- | --- | --- | --- | --- | --- | --- |
|  | Selected variables | Depth |  | Selected variables | Depth |  | Selected variables | Depth |  | Selected variables | Depth |
| Overall | Age | 1.949 |  | Length | 2.441 |  | TNM | 2.302 |  | TNM | 2.262 |
|  | TNM | 2.189 |  | TNM | 2.583 |  | Length | 2.635 |  | Length | 2.308 |
|  | Length | 2.236 |  | Age | 2.597 |  | Age | 2.672 |  | Age | 2.711 |
|  | ***Station.1*** | 2.669 |  | PNLVI | 3.751 |  | RCT | 4.005 |  | PNLVI | 4.129 |
|  | RCT | 2.797 |  | RCT | 4.687 |  | ***Station.16*** | 4.313 |  | RCT | 4.152 |
|  | PNLVI | 2.872 |  | ***Station.8L*** | 4.766 |  | ***Station.8M*** | 4.478 |  | ***Station.1*** | 4.524 |
|  | ***Station.2*** | 2.943 |  | ***Statioin.16*** | 4.920 |  | Sex | 4.488 |  | ***Station.8L*** | 4.553 |
|  |  |  |  |  |  |  | ***Station.8L*** | 4.505 |  | Location | 4.571 |
|  |  |  |  |  |  |  | PNLVI | 4.546 |  | ***Station.16*** | 4.744 |
|  |  |  |  |  |  |  |  |  |  | Sex | 5.428 |
|  |  |  |  |  |  |  |  |  |  | ***Station.8M*** | 5.652 |
|  |  |  |  |  |  |  |  |  |  | ***Station.2*** | 5.732 |
|  |  |  |  |  |  | ^a^Prediction error (OOB)=38.111%; threshold=6.017 | | | | | |
| Disease-free Age | | 1.992 |  | Length | 2.453 |  | TNM | 2.373 |  | TNM | 2.271 |
|  | Length | 2.315 |  | TNM | 2.513 |  | Age | 2.616 |  | Length | 2.354 |
|  | TNM | 2.321 |  | Age | 2.787 |  | Length | 2.658 |  | Age | 2.773 |
|  | ***Station.1*** | 2.907 |  | PNLVI | 3.684 |  | RCT | 2.586 |  | PNLVI | 3.458 |
|  |  |  |  | RCT | 4.716 |  | ***Station.16*** | 4.122 |  | RCT | 4.168 |
|  |  |  |  | ***Station.8L*** | 4.872 |  | PNLVI | 4.348 |  | Location | 4.647 |
|  |  |  |  | ***Station.2*** | 5.022 |  | Sex | 4.561 |  | ***Station.16*** | 4.854 |
|  |  |  |  | ***Statioin.16*** | 5.093 |  | ***Station.8L*** | 4.630 |  | ***Station.8L*** | 4.931 |
|  |  |  |  |  |  |  | ***Station.8M*** | 4.860 |  | ***Station.1*** | 4.950 |
|  |  |  |  |  |  |  |  |  |  | ***Station.8M*** | 5.048 |
|  |  |  |  |  |  |  |  |  |  | ***Staion.2*** | 5.285 |
|  |  |  |  |  |  |  |  |  |  | Sex | 5.286 |
|  |  |  |  |  |  | ^a^Prediction error (OOB)=39.344%; threshold=6.061 | | | | | |

^a^The prediction error rate of out-of-bag (OOB) data and the depth thresholds will change with cases used for modeling; only the indexes for all patients are presented in the table.

The ***bold italics*** indicate the corresponding LN stations were selected. All qualified variables are shown and sorted by the rank of depth.
CE, cervical esophagus; UTE, upper thoracic esophagus; MTE, middle thoracic esophagus; LTE, lower thoracic esophagus PNLVI, perineural lymphovascular invasion; CRT, chemoradiotherapy.
